# Learning to drive in young adults with language impairment

**DOI:** 10.1016/j.trf.2016.07.015

**Published:** 2016-10

**Authors:** Kevin Durkin, Umar Toseeb, Andrew Pickles, Nicola Botting, Gina Conti-Ramsden

**Affiliations:** a University of Strathclyde, School of Psychological Sciences and Health, UK; b The University of Manchester, School of Psychological Sciences, UK; c King’s College London, Institute of Psychiatry, Psychology and Neuroscience, UK; d City University London, Language and Communication Science, UK

**Keywords:** Driving, Young adults, Language impairment

## Abstract

Language impairment (LI) is a common developmental disorder which affects many aspects of young people’s functional skills and engagement with society. Little is known of early driving behaviour in those with this disability. This longitudinal study examines early driving experience in a sample of young adults with LI, compared with a sample of typically developing age-matched peers (AMPs). At age 24 years, significantly fewer participants with LI had acquired a driving licence. A crucial hurdle for those with LI appeared to be the Theory part of the (UK) test. Logistic regression analysis indicated that language ability and a measure of independence at age 17 contributed to the prediction of licence possession at age 24. There was no evidence of differences in traffic violations or accident rates between those with and without LI. There is little evidence that young people with LI are at greater risk on the roads than peers without LI, but some individuals with LI might benefit from support in the course of preparation for driving and in the driving test.

## Highlights

• First study of entry into driving by young people with language impairment (LI).
• Individuals with LI are less likely than peers to have acquired a licence by age 24.
• Language and independence at 17 contribute to the prediction of holding a licence.
• Individuals with LI are at no greater risk of traffic violations/accidents.
• Individuals with LI may benefit from support during the preparation and test phases.

## 1 Introduction

Learning to drive is an important dimension of achieving independence and self-sufficiency for many adolescents and young adults (Barkley & Cox, 2007). It affords or improves access to a larger geographical environment, it expands opportunities for employment and leisure, and it signals adult autonomy. It also places the young person at disproportionate risk of accidents: in most countries, young drivers are the highest risk group for road accidents and fatalities (World Health Organization, 2013). Hence, driving is at once a valuable skill, an indicator of social inclusion, and a source of major public health issues. For these reasons, there is considerable research and policy interest in the adequacy of preparation of young people for participation in road traffic, in the inclusion/exclusion of particular groups of potential transport users (Lamont, Kenyon, & Lyons, 2013), and in the factors that help to predict safe versus risky driver behaviour (Bina et al., 2006, Durkin and Tolmie, 2010, Romer et al., 2014, Shope and Bingham, 2008).

One factor which might be anticipated to bear on young people’s driving-related choices and competence is whether or not they have developmental disabilities. For example, much of the literature on young drivers has focused on those with Attention Deficit Hyperactivity Disorder (ADHD). A large body of evidence indicates that individuals with this condition are at greater risk than non-affected peers of violating traffic safety regulations, receiving citations for driving offences, and having road accidents (Barkley and Cox, 2007, Barkley et al., 1996, Chang et al., 2014, Woodward et al., 2000). The symptoms characteristic of ADHD, including poor attention, impulsivity, hyperactivity and weak inhibitory control (Barkley, 1997) clearly present challenges to driver performance (although the extent of the risk is controversial, cf. Oliver et al., 2015, Vaa, 2014).

Researchers have also begun to address the difficulties of individuals with dyslexia, drawing attention to the fundamental but widely neglected observation that these ‘are transport users, too’ (Lamont, 2009). Lamont and colleagues’ in-depth qualitative analysis demonstrates that the difficulties faced by people with dyslexia with spoken and written language impact on their uses of travel information, presenting obstacles to planning and executing journeys (Lamont et al., 2013). Brachacki, Nicolson, and Fawcett (1995) tested the abilities of adults with dyslexia to differentiate real and false traffic signs and found that the participants with dyslexia were less able to perform the task than were typical adults.

Less attention has been paid to the substantial proportion of young people who have language impairment (LI). These individuals are also transport users and, in many cases, will become drivers at some point. In this study, we present what we believe to be the first investigation of how young adults with LI progress towards becoming drivers.

### 1.1 Language impairment and driving

LI is a disability that is often unnoticed in everyday life yet can have pervasive consequences for the young person concerned. The major clinical symptoms of this disorder are deficits in the comprehension and/or production of language in the context of nonverbal intelligence within the normal range and the absence of any hearing impairment or frank neurological damage (Leonard, 2014). The condition is heterogeneous (different children have different combinations of characteristics) but many individuals with LI have receptive difficulties detecting subtle features of everyday talk, such as the small parts of speech (e.g., plural markers, inflections, past tense expressions) that contribute importantly to the exchange of meaning. Many have expressive difficulties in producing these details and appear stilted in speech. Many have both receptive and expressive difficulties. As a result, young people with LI find it difficult to keep up with conversations and to process complicated instructions. Approximately 7% of children have LI at the point of school entry (Tomblin et al., 1997) and, although it can resolve during the school years, for many LI continues into adolescence and adulthood (Conti-Ramsden et al., 2012, Nippold and Schwarz, 2002).

Why should LI be relevant to the tasks of the novice driver? LI can be expected to bear on transport use for reasons similar to those identified in young people with other, related, developmental disorders. Importantly, it is well-established that there are overlaps and comorbidities among ADHD, dyslexia and LI (Bishop and Snowling, 2004, Newbury et al., 2011, Redmond, 2016). For example, children with LI tend to manifest levels of hyperactivity and emotional difficulties that are significantly higher than those of typical comparison groups (Beitchman et al., 1986, Cardy et al., 2010, St Clair et al., 2011). High proportions of children with LI have reading difficulties (Bishop and Snowling, 2004, Conti-Ramsden et al., 2001, St Clair et al., 2010).

Young people with LI have also been found to lag behind peers with typical development in the achievement of independence in many aspects of daily life (Conti-Ramsden & Durkin, 2008). For example, Conti-Ramsden and Durkin found that virtually all (99%) of the parents of a control sample of typically developing 16-year-olds reported that their children were fully able to look after themselves in respect of everyday life tasks, that their children were able to travel independently (i.e., using public transport), and could organize their own going out; however, the corresponding parental endorsements for 16-year-olds with LI on the same three measures were 64%, 78%, and 62%, respectively. Although using a very different methodology and with a different sample, this dovetails with the findings of Lamont et al. (2013) concerning people with dyslexia who reported that they sometimes needed help executing their travel plans and sometimes, if help was not readily available, avoided or abandoned journeys. This suggests that, as a group, adolescents with LI achieve less familiarity with the daily tasks of transport use and are less equipped with the general level of personal independence that could form the backdrop to a desire to learn to drive.

In sum, the entry barriers to driving – such as learning how to drive, taking driving tests – normally involve listening to instructions, articulating plans, and reading texts and signs; all of these pose potential obstacles to those with language difficulties. Learning to drive is an important developmental task for many young people, but those with LI tend to be delayed in addressing developmental tasks and in achieving autonomy. Taken together, these factors lead to the expectation that entry into driving will be later in young people with LI than in typically developing comparison peers.

### 1.2 The present study

The present study investigates the uptake of driving and the early driving records of a sample of young people with histories of LI, and compares them to a group of peers with typical development. In the light of the above, we focussed on two aspects, each of which has theoretical and practical importance. These are: developmental readiness for entry into driving (i.e., seeking and acquiring a licence, confidence in driving) and safety indicators (i.e., rule violations and accidents). Developmental readiness is of theoretical interest in helping to understand how different abilities – in the present case, language abilities – impact on tasks associated with entry into adulthood. It is also of practical importance, in that access to driving mobility is a measure of social inclusion (Lamont et al., 2013). It would be of concern, for example, if a substantial minority of young people was unwittingly excluded from road use because of language disability if, in fact, there was no relationship between the disorder and driving behaviour.

Safety indicators are of theoretical interest as measures of the real-world consequences of impairments. In respect of adolescents with ADHD, Barkley and Cox (2007) review evidence to suggest that people with this condition manifest impairments in operational driving skills (including reaction time, speed of cognitive processing, attention, concentration, visual scanning, visual-motor integration) and that these deficits contribute to poorer safe driving records and higher accident rates among those with ADHD. Deficits in similar cognitive-perceptual skills have also been identified in children and adolescents with LI (Bishop, 2002, Finneran et al., 2009, Hick et al., 2005, Hill, 2001, Powell and Bishop, 1992). However, it is unknown whether there is an association between LI and driving safety problems. Again, the practical import of this question is that it speaks to community safety needs and to whether additional training and/or treatment would be advisable before allowing young people with LI to drive. One purpose of the present investigation was to provide the first body of relevant evidence.

We predicted that, compared to their typically developing peers, a smaller proportion of young people with LI would learn to drive and that, among those who did drive, confidence in driving would be lower than in their typically developing counterparts.

The basis for predictions concerning actual safety indicators is less straightforward. On the one hand, unlike adolescents with ADHD, there is no evidence to suggest that adolescents with LI are any more prone to risk-taking or antisocial behaviour than is normative among the typically developing population. On the other hand, as tasks such as reading signs and responding to instructions are potentially difficult for those with LI and, as there is evidence of cognitive-perceptual deficits associated with the disorder, it is possible that these characteristics could impact on decision making and errors in traffic; in turn, this could result in higher than normative accident rates. This is an open empirical question, and we aimed to provide preliminary evidence.

We took note also that Lamont et al. (2013) reported that many adults with dyslexia found satellite navigation (sat nav) technology extremely beneficial. This is of interest to the present investigation because comprehension of real-time language is by definition difficult for many of those with LI. Using sat nav entails coordinating ‘live’ verbal instructions with on-screen maps and symbols and relating all of this information to the driver’s current location, goals and strategic plans. As well as their language difficulties and associated problems with online processing, adolescents with LI experience greater difficulties with using computer technologies and higher levels of computer anxiety than do adolescents without LI (Conti-Ramsden et al., 2010, Durkin et al., 2010). Hence, on the one hand, those with a related disability (dyslexia) appear to fare well with this technology (or, at least, perceive it as very helpful) but, on the other hand, there are reasons to anticipate that it could be difficult and even threatening for those with LI. In relation to use of sat navs, we aimed to obtain self-report information which could guide future observational and experimental research.

## 2 Method

### 2.1 Ethics

The study reported here received ethical approval from The University of Manchester, UK. Participants and their parent/legal guardian (for the parental questionnaires) provided written informed consent.

### 2.2 Participants

Participants were recruited as part of the Manchester Language Study, a large-scale longitudinal research programme that began when the children with LI were 7 years of age (Conti-Ramsden and Botting, 1999, Conti-Ramsden et al., 1997). At 16/17 years of age a comparison group of young people (age-matched peers, AMPs) were also recruited. These participants had no history of special educational needs or speech and language therapy provision. For both groups of participants, not all participants who took part at age 24 took part at age 16/17.

#### 2.2.1 Young people with LI

The initial cohort of 242 children with LI originally consisted of 186 boys (77%) and 56 girls (23%), and was recruited from 118 language units across England. They represented a random sample of 50% of all 7-year olds attending language units. Language units are specialist resource classes for children who have been identified with primary language difficulties, which are attached to regular schools. Although attrition occurred, this was partly due to funding constraints at follow-up stages of the study. For this study, analysis was conducted on data collected at ages 16/17 (M 16.5, SD 0.4 years) and 24 years (M 24.4, SD 0.7 years). At age 16/17, there were 80 young people with LI (56 males and 24 females) and at age 24, there were 84 young people with LI (56 males and 28 females). From the 84 young people with LI at age 24, 57 provided data at both time points.

#### 2.2.2 Comparison peers

The comparison group of peers was selected to be of similar age, similar geographical area, and similar socioeconomic background as the young people with LI. The comparison group was of a similar age to the sample with LI at each time point (16/17: M 16.4, SD 0.4 years, 24: M 24.1, SD 0.9 years). At age 16/17, there were 85 participants in the AMP group (53 males and 32 females) and at age 24, there were 88 (49 males and 39 females). Participants at age 16/17 came from similar geographical locations as the sample with LI. The comparison group came from the same schools as the participants with LI as well as additional targeted schools to ensure a similar urban versus rural geographical distribution in both groups. In addition, participants in the comparison group were sampled from selected demographic areas in order to ensure comparison peers came from a broad range of socioeconomic backgrounds, similar to participants with a history of LI. The LI and the comparison groups did not differ on household income at age 16/17 years (*χ*^2^(10, *N* = 145) = 9.32, *p* = .501) nor personal income at age 24 years (*χ*^2^(5, *N* = 131) = 7.38, *p* = .194). For ease of reading, the comparison group is referred to as age-matched peers (AMP). From the 88 young people in the AMP group, 53 provided data at both time points. As expected, language, PIQ, and reading profiles were different for the groups at both time points (Table 1).

### 2.3 Materials

#### 2.3.1 Language measures

The Clinical Evaluation of Language Fundamentals (CELF-IV^uk^, Semel, Wiig, & Secord, 2006) was used at both time points. Neither group reached ceiling levels on this assessment which is normed up to age 21;11 years; therefore, the CELF-IV was deemed the best fit assessment for our cohort at 24 years of age. The Word Classes subscale for receptive language and Recalling Sentences subscale for expressive language were used at both time points. A core language score at age 16/17 was also derived using four subsets of the CELF-IV in accordance with the scoring manual: Recalling Sentences, Formulated Sentences, Word Classes 2, Word Definitions.

#### 2.3.2 Non-verbal IQ

The Wechsler Abbreviated Scale of Intelligence (WASI, Wechsler, 1999) Performance subscale was used at both time points.

#### 2.3.3 Reading measures at age 16/17

The Sight Word Efficiency Subscale of the Test of Word Reading Efficiency (TOWRE; Torgesen, Wagner, & Rashotte, 1999) was used. During this assessment, participants were required to identify the maximum number of words within set time duration.

#### 2.3.4 Independence measure at age 16/17

The parent-report independence questionnaire (Conti-Ramsden & Durkin, 2008) was used. Questions covered self-help skills (e.g., looking after oneself), ability to carry out necessary tasks for every living (e.g., using the telephone), and the ability to carry out activities outside the home (e.g., travelling independently, going out on their own or with friends). Responses to the 11 questions were summed to create an overall score between 0 and 11. Higher scores indicated greater independence. The Cronbach’s *α* for this administration of the scale was .77.

#### 2.3.5 Driving related measures at age 24

*A driving related behaviours questionnaire* collected basic information about driving experience in four categories: general driving (5 items; e.g., ‘Can you drive a car?’), theory test (6 items; e.g., ‘Have you taken the Theory Test?’), practical test (6 items; e.g., ‘Did you find the Practical Test easy or hard?’), and use of satellite navigation system (3 items; e.g., ‘Do you use a sat nav?’).

*A driving confidence scale* was adapted from previous measures of driving confidence (Baldock et al., 2006, Parker et al., 2001). This assessed the individual’s confidence in a range of driving contexts. Example items include: ‘How confident are you in your ability to drive alone?’, ‘… drive on the motorway?’, ‘… drive at night?’. Responses to each item were on a 5-point scale (1 ‘Not at all confident’, 5 ‘Very confident’). Scores ranged from 8 to 40; a higher score indicated higher confidence (Cronbach’s *α* = 0.8).

*Safety indicators* were assessed using an adapted version of the *driving history interview* (Barkley, Murphy, Dupaul, & Bush, 2002). This collects self-reports of violations of traffic rules and experience of accidents. Example items include: ‘How many times have you received a speeding ticket?’, ‘… driven without insurance’, ‘… been determined to be at fault in an accident?’ Note that it is possible to give answers greater than 1 for any question (e.g., for the latter question, answers ranged from 0 to 6). Total scores from the 11 items were summed to create an overall safety indicators score. A higher score indicated more problematic driving behaviours.

### 2.4 Procedure

The participants were interviewed face-to-face on the measures described above as part of a wider battery. Interviews took place in a quiet room, wherever possible with only the participant and a trained researcher present. Standardized assessments of nonverbal and verbal skills were administered in the manner specified by the test manuals. During the interview, the items were read aloud to the participants. The items and response options were also presented visually to ensure comprehension.

### 2.5 Statistical analysis

All statistical analyses were conducted in Stata/SE 13.1 (StataCorp, 2013) and a two-tailed significance level of *p* = .05. *P* values are reported up to 3 decimal places and test statistics, means and standard deviations to 1 decimal place. Chi-squared tests were used to test for differences in binary responses and independent samples *t*-tests were used to test for differences in continuous responses. Using predictors measured at age 16/17 years, logistic regression analysis was conducted to calculate the likelihood of holding a full driver’s licence by age 24 years. In the cross-sectional analyses, data from all participants was used. For the longitudinal analysis, only participants who provided data at both time points were included.

## 3 Results

### 3.1 Engagement with driving

Comparisons between young people with LI and AMPs for questions relating to general driving, the theory test, and the practical test are shown in Table 2.

### 3.2 General driving

In terms of general driving, fewer young people with LI stated that they could drive a car and fewer had a full driving licence compared to their AMPs. For those who could not drive, there were no differences between the young people with LI and their AMPs in their desire to drive a car or their intention to learn to drive. These comparisons are shown in Table 2.

Of those who had learned to drive or were learning to drive, there was no difference between the groups in terms of the age at which they started to do so, which was 18.1 years (SD 1.8) in the LI group, and 18.1 years (SD 1.6) in the AMPs, *t* (115) = 0.1, *p* = .96.

### 3.3 Theory test

Fewer young people with LI had taken the theory test. Of those who had taken it, fewer young people with LI had passed it. More young people with LI took more than one attempt to pass their theory test, and more of the young people with LI rated the theory test as either hard or very hard. When participants were asked what they found difficult about the theory test, young people with LI selected reasons such as reading the instructions, reading the questions, the time limit, hazard perception, remembering the information, and understanding the questions. The AMPs did not select reading the instructions, reading the questions, and the time limit as reasons for their difficulty. Similarly to the young people with LI, however, some experienced difficulties with the hazard perception test, remembering the information, and understanding the questions.

### 3.4 Practical test

Fewer young people with LI had taken the practical test compared to their AMPs. However, of the young people with LI who had taken the practical test, the pass rate was not different to AMPs, and it did not take them more attempts than their AMPs. For those who failed, there was no difference between the young people with LI and AMPs in terms of how many times they had failed. For all participants (those who had passed or failed the practical driving test), there was no difference between young people with LI and their AMPs in their rating of the practical test as either hard or very hard. When participants were asked what they found difficult about the practical test, young people with LI selected difficulties such as understanding directions/instructions from the examiner, technical questions, and nerves. The AMPs selected difficulties such as nerves and the pressure of the test.

### 3.5 Use of satellite navigation (sat nav)

There was no difference between young people with LI and their AMPs in the use of a sat nav whilst driving or their perceived difficulty when using the sat nav. None of the young people with LI reported difficulty using the sat nav and three of the AMPs reported difficulties: understanding the directions given was the main difficulty reported.

### 3.6 Driving confidence

There was no difference in self-reported driving confidence between the two groups. The driving confidence means were 33.8 (SD = 3.9) for LI and 34.2 (5.4) for AMPs, *t* (92) = −.04 *p* = .710.

### 3.7 Safety indicators

There were no differences in the safety indicators between the groups. The means and SDs were 4.1 (12.6) for LI and 4.0 (9.5) for AMPs, *t* (97) = .08 *p* = .935.

### 3.8 Predicting possession of a full driving licence at age 24

Significantly fewer young people with LI had obtained a driving licence by age 24, compared to their AMPs (Table 2). To investigate the extent to which language, reading and independence influenced the likelihood of attaining a driving licence in young people with LI, a logistic regression was run in which the outcome variable was possession of a driving licence at age 24, and the predictors were language, reading, and independence, all measured at age 16/17. Thus, the analysis tests longitudinally to what extent characteristics identifiable at around the age the young person becomes eligible to learn to drive help to predict the likelihood of gaining a licence within the next few years. The zero order correlations among the variables are presented in Table 3, and the results of the logistic regression are presented in Table 4.

The analysis revealed a significant overall effect. Language and independence scores were identified as contributing significantly. This pattern of results was the same when controlling for PIQ and when controlling for gender. The same analysis was conducted for the group of AMPs but no significant effects were obtained.

## 4 Discussion

This is the first study of the early driving-related choices and experiences of young people who have grown up with language impairment. Because of previous evidence that a proportion of adolescents with LI are lacking in some of the indicators of personal independence and mobility, we expected that by early adulthood there would be lower levels of participation (obtaining a licence) in this group than in a group of age matched peers. This proved to be the case, with only 43% of our sample of young adults with LI having obtained a full driving licence, compared to 75% of those of the same age, geographical and socioeconomic backgrounds without LI. Fewer participants with LI had taken the theory component and, of those who had taken it, a smaller proportion had passed it. More participants with LI required more than one attempt to pass their theory test, and more participants with LI rated the theory test as either hard or very hard.

With respect to the second stage of the test, fewer participants with LI had taken the practical component, compared to AMPs. However, of the participants with LI who had taken the practical test, the pass rate was not different to that for the comparison group, and they did not require more attempts than the comparison group to pass it. For those who failed, there was no difference between the participants with LI and those without LI in terms of how many times they had failed. Among those who had attempted the practical driving test (whether they passed or failed), there was no difference between the LI group and the comparison group in their rating of this component as hard or very hard.

**Fig. 1:**
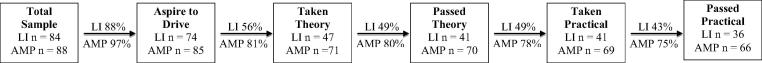
Progress through key stages of attaining a driving licence. Note: Figures above (LI) and below (AMP) arrows indicate the percentage of the total sample who had progressed from the stage on the left to the subsequent stage.

A diagrammatic summary of progress through the key stages of pursuit of a driving licence is presented in Fig. 1. Some participants indicated no interest in learning to drive, so the diagram focuses on those whose aspirations to become a driver had already been realised, were in progress, or were expressed as a desire to be able to do so. Together, these are labelled as ‘Aspire to drive.’ Slightly more participants in the AMP group aspired to drive, but this was not a significant difference; the question of interest, then, is how the respective groups fared in the period under study.

These findings suggest that a crucial hurdle for young people with LI learning to drive in the UK is the theory test. A proportion of those with LI have not attempted this stage and, of those who have, high numbers (relative to those without LI) appear to have experienced difficulties. Because they are not eligible to proceed to the practical test until the theory test is passed, a smaller proportion of those with LI reach the practical stage - but, if they do reach it, they are no more likely to fail.

The participants’ own indications of what they found difficult about the theory test were consistent with the interpretation that the language and information processing features of the task were problematic for them. Participants with LI selected reasons such as reading the instructions, reading the questions, the time limit, hazard perception, remembering the information, and understanding the questions. The AMPs did not cite reading the instructions, reading the questions, and the time limit as reasons for their difficulty. However, similarly to the LI participants, some reported difficulties with hazard perception, remembering the information, and understanding the questions.

Difficulties indicated in relation to the practical test add to this picture. Participants with LI selected difficulties such as understanding directions/instructions from the examiner, technical questions, and nerves. The AMPs selected difficulties such as nerves and the pressure of the test.

A strength of this study was that we had longitudinal data available, enabling us to test prospectively the relationship between ability measures collected at age 16/17 and driving outcomes reported by age 24. The results of the logistic regression analyses provide further support for the inference that language-related difficulties bear on the early orientation towards driving in those with LI. Specifically, poorer language scores at age 17 and lower parent-rated independence scores at age 17 each contributed to the prediction of possession of a driving licence at age 24. This is consistent with a broad pattern of evidence that, throughout development, poorer language abilities are associated with a range of less favourable outcomes (Clegg et al., 2005, Durkin and Conti-Ramsden, 2010, Johnson et al., 2010, Mawhood et al., 2000). Independence (as rated by parents) was the stronger predictor. Taken together, these findings suggest that the impact of language impairment on learning to drive is part of a broader burden, such that those with LI find many life tasks more difficult than do their peers without LI.

We did not find evidence of an additional effect of literacy ability on driving preparation. Note that language ability and literacy are strongly associated (Table 3) and the latter does not add to the analysis once the former is taken into account. Although it appears plausible that individuals who struggle more with aspects of reading will be deterred from taking tests that entail a lot of written materials, it may be that the motivations to learn to drive are sufficient to overcome this potential barrier. Our study did not examine effects of reading ability in timed contexts – for example, during either component of the driving test or in actual or simulated road conditions. These remain issues for future research.

It is important to emphasize that we are presenting data here that relate to a particular stage of the participants’ lives: at the time when we solicited information on their driving qualifications and experiences, all were young adults, in their early 20s. It is perfectly possible that some of those who have not yet sought licences will do so in the future. Nevertheless, it is clear that members of this group (LI) are more likely to experience delay in learning to drive, relative to peers with typical development.

It is possible that later entry into the world of driving is adaptive for some individuals with LI. In their late teens/early 20s, they may not feel ready for learning to drive but the benefits of being able to do so may subsequently become more compelling and developmental changes in their desire for independence might in due course lead to changes in behaviour. The period taken into account in this study (17–24 years) is a time during which many young people undergo substantial changes in their attitudes towards driving and their involvement in risky road behaviours (Møller & Haustein, 2013); it could be argued (and accident rates and the pricing of insurance for young drivers underscore the point) that later entry is safer for most.

There was a slight, non-significant, tendency for those with LI to be less likely to use sat nav than were those without LI (58% vs. 71%, respectively). Among those who did use the technology, it was not perceived as an area of difficulty for either participants with LI or the AMPs. This is consistent with the self-reports of people with dyslexia that they often found sat nav beneficial (Lamont et al., 2013). It could be that there may be negative features of sat nav (e.g., distractions from the road, inefficient directions) that occur but are not fully recognised or acknowledged by drivers. Even so, there is no evidence from the present study that those with LI are more, or less, susceptible to any such consequence, and there was no indication that they were more vulnerable to misunderstandings or confusions about directions than were other drivers of their age.

One limitation of the present study is that it did not obtain discursive data. It would be advantageous in future work to adapt qualitative techniques, such as those of Lamont et al. (2013), to address participants’ own perceptions of what they find helpful or unhelpful. Such research could also examine more closely how people with LI use technologies in areas found to be difficult for those with dyslexia, such as journey planning and rehearsal (Lamont et al., 2013). The possibility emerges from the present findings that sat nav technology, generally designed to be user-friendly and facilitative, is indeed useful to those with LI, though more extensive research in road or simulated road conditions would be desirable.

In relation to policy, no evidence emerges here to suggest that young people with LI are a more problematic group of drivers than those without LI (i.e., there was no between-groups difference in safety indicators scores). A direct comparison with young drivers with other developmental disorders, such as ADHD and/or dyslexia, was beyond the scope of this study. We do know from other research that those with ADHD are at significantly greater risk of violations and accidents than peers without the condition (Barkley & Cox, 2007). In the case of individuals with ADHD, one possible intervention is to discourage early entry to driving. Overall, our findings indicate that the policy implications in respect of those with LI are not that the group or their families need to be counselled against learning to drive; instead, the salient issues for these young people relate to inclusiveness and support (which is similar to needs identified in transport users with dyslexia, Lamont et al., 2013). Some young people with LI are likely to lag their peers in terms of when, or if, they seek and obtain driving licences. This could reflect a sense of discomfort with language-related aspects of the training and test procedures and/or lack of readiness for independence. This presents issues for those who might influence the young person’s decision making in this area. For example, some may convey (unintentionally or overtly) guidance that learning to drive should be postponed. In some cases, it may indeed be appropriate to wait until the young person feels ready; in others, it may be that specific support with the language and reading requirements could help them to overcome the first stage of the formal test and they may then be on a par with other aspirant drivers. The UK test does aim to offer facilities to help individuals with disabilities and special needs (https://www.gov.uk/practical-driving-test-for-cars/special-needs). These include allowing extra time for persons with dyslexia or other reading difficulties. However, arrangements have to be requested and it is not known to what extent young people with LI make such requests; furthermore, given that this hidden disability is not widely known in the lay community, it is not clear that the basis for requests would be understood by all test officials.

### 4.1 Future research

Examining performance under simulated driving conditions has proven informative in terms of identifying the difficulties of young drivers with ADHD (Barkley & Cox, 2007). This may prove to be the case also with young people with LI. It should be borne in mind that, from the present findings, there is less evidence to warrant regarding young people with LI as a higher risk group, compared to their peers without LI. Nevertheless, it is possible that simulations can illuminate any aspects of the driving task that may be more difficult for this group. Examples might include road conditions in which the requirement to read signs or other textual road instructions and make sign-related decisions is high, conditions in which signs/textual warnings are partially obscured by ‘natural’ events such as adverse weather or presence of other vehicles, and conditions in which signs are passed at relatively high speed.

Of course, it is not possible to control for the order of administration of the two stages of the driving test (theory and practical) in the real world, but this could be attempted in simulations. This would provide fuller evidence on whether the theory test is a key hurdle for many with LI and this in turn would guide the focus of intervention strategies and counselling.

Research is also needed to extend the present enquiry into other driving environments (i.e., different nations). While many young people do seek to learn to drive in the UK, the relative density of the population in this and other European countries, together with the availability of extensive public transport networks, make it less essential to drive than in many parts of North America, South America, Australia or New Zealand. It is also possible that there are urban versus rural differences (some cities may provide good public transport, some country areas may have little). Thus, it is possible that in some locations there are strong practical reasons for young people to seek licences early; this may impose additional pressure on some young people with LI who may not yet have developmental readiness for this particular task.

Finally, international evidence over the last couple of decades points to substantial and ongoing reductions in the proportions of contemporary young people who are learning to drive, compared to earlier generations (Delbosc and Currie, 2013, Noble, 2005, Sivak and Schoettle, 2016). The reasons for this are multifaceted, and include rising costs (of lessons, vehicles, fuel, insurance), changing social patterns (such as increased peer contact via social media), but one factor highlighted by Noble (2005) was that the introduction of theory tests had led to approximately one year’s delay in the progress of young people in general towards obtaining licences. The present findings resonate with this point, suggesting that those with LI are particularly likely to be affected by this requirement. We are indebted to an anonymous reviewer for pointing out that it is also possible that the wider contextual dynamics may influence the behavioural decisions of those with LI (for example, by making it more acceptable in the peer community not to seek or have acquired a licence). Hence, it would be valuable in future large sample studies to take account of developmental disabilities such as LI (but also ADHD, dyslexia, and other conditions) to examine whether these young people contribute disproportionately to the numbers of the non-licensed or later-licensed. This is important not simply for comparison purposes but because length of driving experience is a predictor of driving skill (Mayhew et al., 2003, Wikman et al., 1998). In sum, entry to driving is challenging for young people with LI and, as a group, they do not all proceed at the same rate as AMPs. This study indicates that, as anticipated, the difficulties of those with LI tend to arise in relation to the language/textual demands of learning and being tested. In the UK, the theoretical test appears to be the primary locus of difficulties. For those with LI who overcome this hurdle, there is no evidence here that they begin their driving careers as more risky or more rule violating than peers without LI; contrary to expectations, those who do learn to drive and possess licences are no less confident in driving. The main policy implication of these findings is that, to ensure that young people with LI experience the same opportunities as other young people to achieve autonomous road use, it would be desirable to inquire specifically about any language, reading or communication needs young people may have. With such information the facilities to help individuals with disabilities and special needs can be extended to provide language support and to consider where testing procedures might be adapted to accommodate language needs.

## Figures and Tables

**Table 1:** Mean (SD) language and performance IQ scores by group and age.

	Aged 16/17 years		Aged 24 years	
	LI	AMP	LI	AMP
Receptive language	76.6 (18.0)	100.1 (11.8)	83.5 (18.6)	106.2 (8.9)
Expressive language	67.3 (14.8)	96.3 (14.1)	70.6 (15.6)	99.5 (15.4)
PIQ	93.7 (15.3)	106.5 (10.7)	98.8 (15.8)	111.93 (10.3)

**Table 2:** Driving related behaviours by group.

Item	LI *n* = 84	AMP *n* = 88	Test statistic	*p*
*General driving*				
Can drive a car (regardless whether they have a driving licence)	58.3% (49)	80.7% (71)	*χ*^2^(1, *N* = 172) = 10.2	.001
If does not drive, would like to drive car	71.4% (25)	70.6% (12)	*χ*^2^(1, *N* = 52) = 0.0	.950
Has full driver’s licence	42.9% (36)	75.0% (66)	*χ*^2^(1, *N* = 172) = 18.4	<.001
If does not have a full licence, learning or intending to learn	60.4% (29)	77.3% (17)	*χ*^2^(1, *N* = 70) = 1.9	.168
*Theory test (this section completed only by those with full/provisional licence)*				
Taken the theory test	56.0% (47)	80.7% (71)	*χ*^2^(1, *N* = 172) = 12.2	<.001
If taken, passed theory test	87.2% (41)	98.6% (70)	*χ*^2^(1, *N* = 118) = 6.5	.011
If passed theory test, how many times taken?	1.9 (1.2)	1.3 (0.7)	*t*(57) = 2.7	.010
If not passed theory test, how many times taken? (NB only 1 AMP)	2.7 (1.0)	2 (0)	n/a	n/a
If taken theory test, difficulty of theory test (1 Very Hard, 5 Very Easy)	3.2 (1.1)	3.8 (0.9)	*t*(115) = −3.0	.004
*Practical test (this section completed only by those with full/provisional licence)*				
Taken practical test	48.8% (41)	78.4% (69)	*χ*^2^(1, *N* = 172) = 16.3	<.001
If taken, passed practical test	87.8% (36)	95.7% (66)	*χ*^2^(1, *N* = 110) = 2.3	.125
If passed practical test, how many times taken?	2.2 (1.6)	1.8 (1.1)	*t*(53) = 1.5	.130
If not passed practical test, how many times taken?	2.2 (0.8)	2.3 (1.2)	*t*(6) = −0.2	.854
If taken practical test, difficulty of practical test (1 Very hard, 5 Very easy)	3.1 (1.0)	3.2 (1.0)	*t*(107) = −0.1	.940
*Use of satellite navigation (this section only completed by those with full/provisional licence)*				
Use sat nav	58.3% (21)	70.8 (46)	*χ*^2^(1, *N* = 101) = 1.6	.205
Difficulty of sat nav use (1 Very Hard, 5 Very Easy)	4.1 (0.6)	4.2 (0.9)	*t*(57) = −0.4	.683

For binary items, *χ*^2^ tests are reported and the figures shown are the% answered ‘yes’, with actual numbers in brackets. For continuous scales, *t*-tests are reported, figures shown are means (SDs).

**Table 3:** Correlation matrix for variables of interest at age 16/17 for young people with LI.

	1	2	3
1. Language	1		
2. Reading	0.6⁎⁎⁎	1	
3. Independence	0.1	0.1	1

⁎⁎⁎ <.001.

**Table 4:** Binomial logistic regression model to predict driving licence possession for LI sample.

	*β*	SE *β*	Wald’s *χ*^2^	*df*	*p*	OR	Pseudo *R*^2^
Overall model			13.75	3	.003		.17
*Predictor*							
Language	.06	0.02	2.36	1	.018	1.05	
Reading	-.05	0.04	−1.31	1	.191	.95	
Independence	.34	0.16	2.19	1	.029	1.41	
Constant	−2.73	2.53	−1.08	1	.280	n/a	
